# Combined use of EUS, ESD, and laparoscopic techniques in the diagnosis and treatment of a mucinous adenocarcinoma of the ascending colon originating from the submucosal layer: a case report

**DOI:** 10.3389/fphys.2026.1745668

**Published:** 2026-04-10

**Authors:** Zongyao Liu, Yanling Li, Rong Tan, Ainizhati Abudusaimaiti, Shengjuan Hu, Ximei Li

**Affiliations:** 1 The Third Clinical Medical College, Ningxia Medical University, Yinchuan, Ningxia Hui Autonomous Region, China; 2 Department of Gastroenterology, People’s Hospital of Ningxia Hui Autonomous Region, Ningxia Medical University, Yinchuan, Ningxia Hui Autonomous Region, China; 3 Department of Pathology, People’s Hospital of Ningxia Hui Autonomous Region, Ningxia Medical University, Yinchuan, Ningxia Hui Autonomous Region, China; 4 Ningxia Clinical Research Institute, People’s Hospital of Ningxia Hui Autonomous Region, Yinchuan, Ningxia Hui Autonomous Region, China

**Keywords:** colon cancer, endoscopic submucosal dissection (ESD), endoscopic ultrasound (EUS), laparoscopic resection, mucinous adenocarcinoma, submucosal tumor

## Abstract

This report describes a 53-year-old woman admitted for evaluation of a space-occupying lesion in the ascending colon detected one year earlier and recent right lower quadrant pain. Initial colonoscopy revealed a 2.0 × 2.0 cm submucosal elevated lesion with surface ulceration near the ileocecal valve. Biopsy showed inflammatory mucosa with abundant mucin but no malignancy, and CT revealed no definite abnormalities. One year later, the lesion enlarged to 3.5 × 3.0 cm with increased ulceration. Endoscopic ultrasound (EUS) demonstrated a hypoechoic submucosal mass, and endoscopic submucosal dissection (ESD) was performed. During ESD, a jelly-like substance raised suspicion of a mucinous neoplasm, leading to laparoscopic resection of the ileocecal region and ascending colon. Pathology confirmed mucinous adenocarcinoma originating from the submucosal layer. This rare presentation differs from typical colorectal cancers that arise from the epithelial layer. The combined use of EUS, ESD, and laparoscopic surgery facilitated accurate diagnosis and effective treatment.

## Introduction

Colorectal carcinoma may occasionally present as a lesion resembling a submucosal tumor (SMT), which can lead to significant diagnostic uncertainty (Nakajima et al., 2005). In such cases, the overlying mucosa may remain intact, and superficial endoscopic biopsies frequently yield nondiagnostic results. Mucinous adenocarcinoma, characterized by abundant extracellular mucin production, may demonstrate this atypical growth pattern and obscure the true invasive nature of the tumor (Hugen et al., 2016).

Endoscopic ultrasound (EUS) plays an important role in evaluating gastrointestinal protruding lesions by determining the layer of origin and internal echo characteristics, and has been shown to have good diagnostic accuracy for submucosal lesions (Polkowski et al., 2012). It is particularly useful in distinguishing intramural lesions from extrinsic compression. However, when colorectal carcinoma exhibits an SMT-like growth pattern, EUS findings may not definitively differentiate malignant tumors from true submucosal neoplasms.

Endoscopic submucosal dissection (ESD) has been widely adopted for selected colorectal lesions because it enables en bloc resection and provides an appropriately oriented specimen for accurate histopathological evaluation (Makino et al., 2015). When a lesion is presumed to be a submucosal tumor, ESD may serve both diagnostic and therapeutic purposes (Abulawi et al., 2025). Nevertheless, unexpected intraoperative findings may necessitate immediate modification of the treatment strategy.

Here, we report a rare case of mucinous adenocarcinoma of the ascending colon presenting as an SMT-like lesion, highlighting the diagnostic challenges and emphasizing the importance of integrating EUS evaluation with timely surgical management.

## Case reports

**Figure 1:**
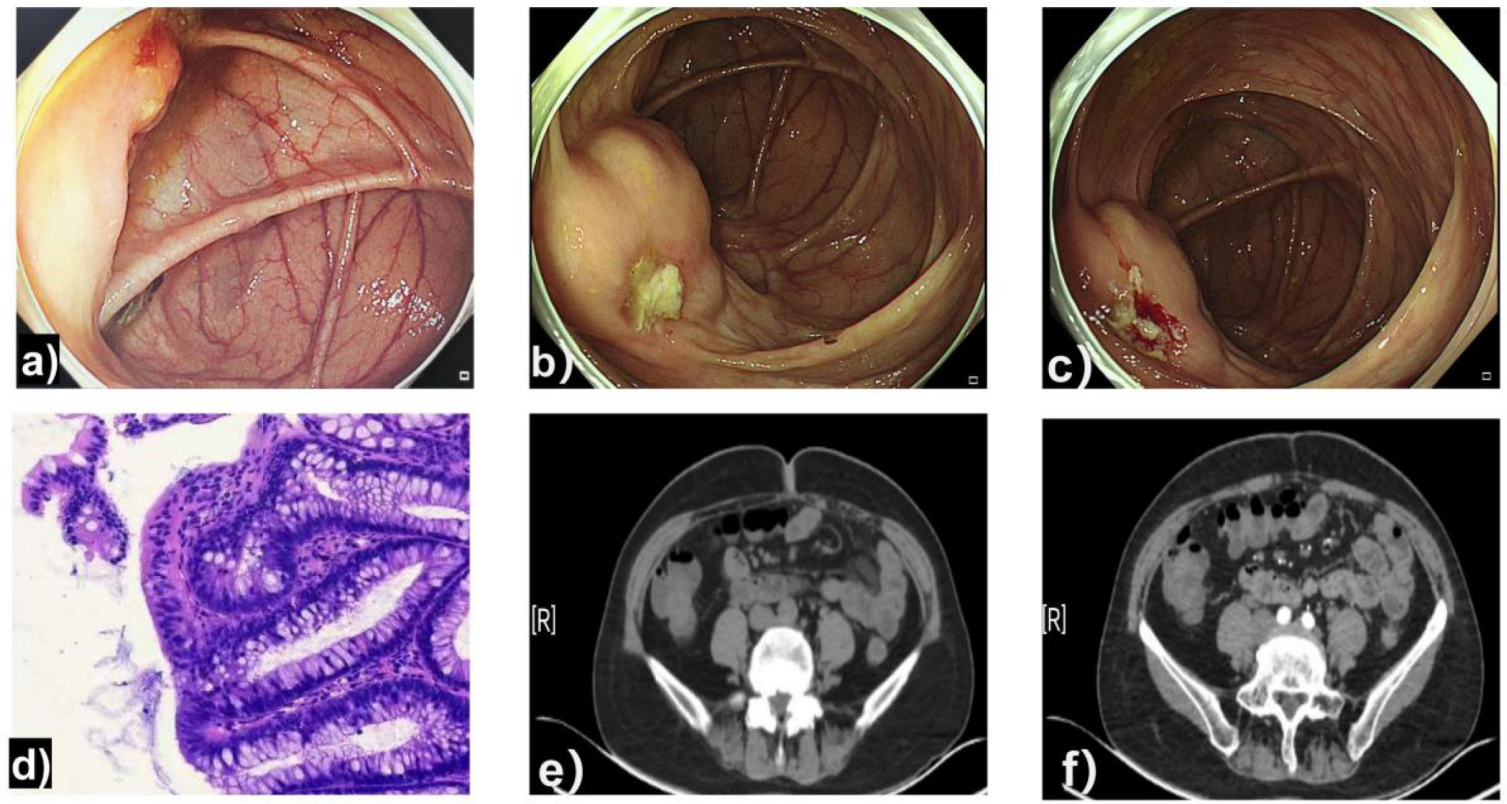
Baseline findings from the initial examination one year prior to admission. **(a–c)** Colonoscopy identifies a submucosal elevated lesion (approx. 2.0 × 2.0 cm) with surface ulceration near the ileocecal valve. **(d)** Biopsy (H&E): Mucosa with inflammation and extracellular mucin. **(e, f)** Abdominal CT scans (plain and contrast-enhanced) show no definite lesion in the corresponding region.

A 53-year-old female patient was admitted to our hospital. One year prior, a surveillance colonoscopy (**Figures 1a–c**) revealed a submucosal elevated lesion, approximately 2.0 × 2.0 cm in size, located in the ascending colon about 2 cm from the ileocecal valve. An ulcer was observed on the lesion surface, from which one biopsy specimen was obtained (**Figure 1c**). Pathological examination of the ulcer site biopsy (**Figure 1d**) showed fragmented colonic mucosal tissue with acute and chronic inflammation, accompanied by abundant mucinous material. Within this mucus, numerous plasma cells and fibroblasts were scattered in focal distributions, along with fragmented glandular structures and cells with eosinophilic cytoplasm. Contrast-enhanced computed tomography (CECT) of the abdomen (**Figures 1e, f**) revealed no significant abnormalities in the ileocecal region or ascending colon. Due to the indeterminate nature of the lesion, the managing physician recommended a follow-up colonoscopy in 3 months to monitor its progression. However, the patient did not comply with this recommendation and did not undergo a repeat colonoscopy during the following year.

**Figure 2:**
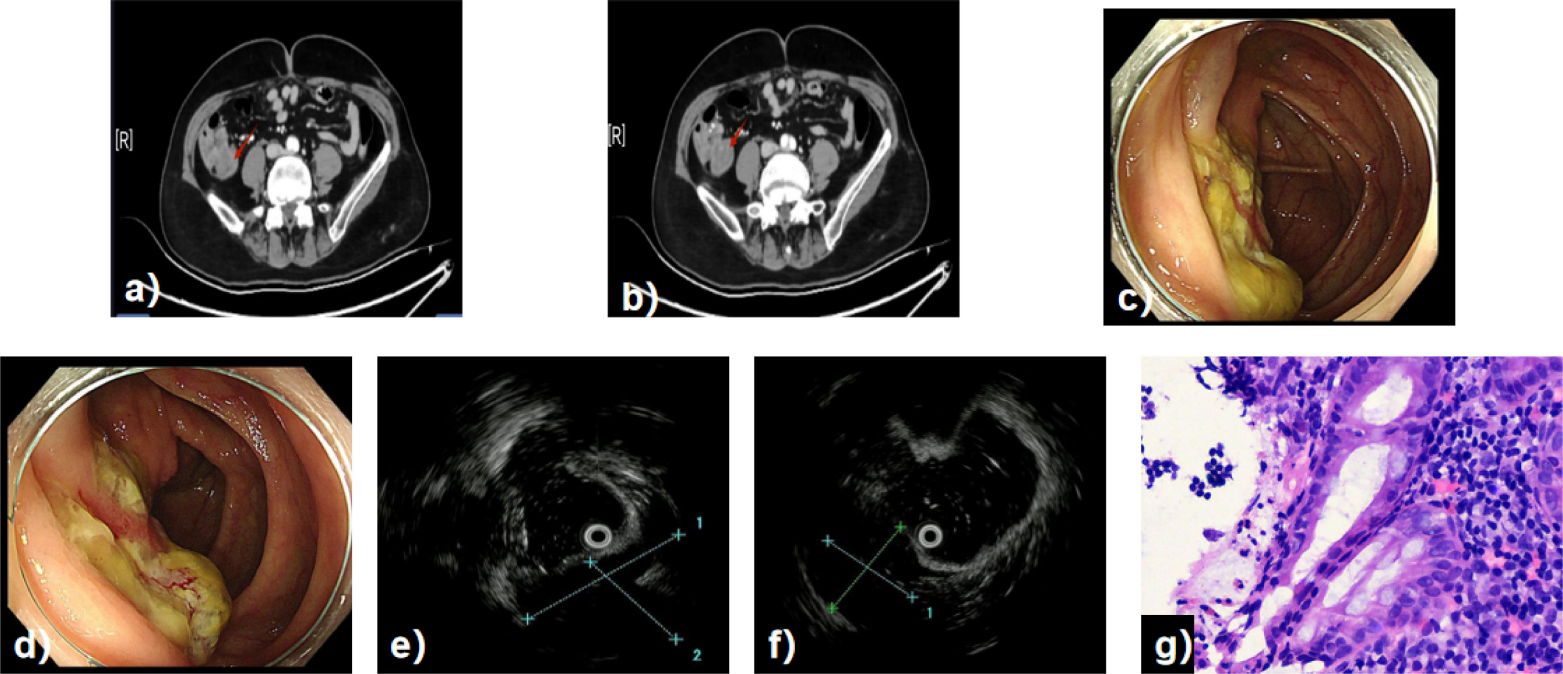
Diagnostic findings at admission. **(a, b)** CT imaging now reveals a heterogeneous mass in the previously unremarkable ileocecal region. **(c, d)** Colonoscopy shows significant enlargement of the lesion to 3.5×3.0 cm, with a firm texture and surface exudate. **(e, f)** EUS localizes the round, hypoechoic mass to the submucosal layer, confirming its mural origin. **(g)** Biopsy (H&E): Mucosa with inflammation.

Two months prior to the current admission, the patient experienced intermittent, dull pain in the right lower quadrant without any identifiable trigger. A repeat colonoscopy showed that the lesion had enlarged to approximately 3.5 cm, felt hard on tactile inspection with the endoscope tip, and exhibited an enlarged surface ulcer. Physical examination upon admission revealed tenderness in the right lower quadrant without rebound tenderness. Tumor markers, including CEA, CA19-9, and AFP, were within normal limits. A repeat abdominal CECT (**Figures 2a, b**) showed heterogeneous density in the ascending colon near the ileocecal region. The following examinations were performed after admission: White-light colonoscopy (**Figures 2c, d**) identified a protruding lesion measuring about 3.5 × 3.0 cm near the ileocecal valve. The lesion surface was covered with copious purulent and bloody exudate. Three depressed areas with purulent covering were noted surrounding the lesion, and two biopsies were taken from these areas. EUS (**Figures 2e, f**) revealed a round, hypoechoic mass originating from the submucosal layer, with a cross-sectional area of approximately 25 × 26 mm. The remainder of the colon, including the hepatic flexure, transverse colon, splenic flexure, descending colon, sigmoid colon, and rectum, appeared normal. Pathological examination of the biopsies (**Figure 2g**) indicated chronic active inflammation of the colonic mucosa with hyperplastic polyp formation and focal erosion.

**Figure 3:**
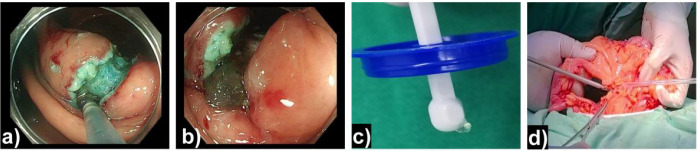
ESD findings and surgical management. **(a–c)** ESD: Mucosal incision revealing gelatinous material. **(d)** Laparoscopic view of the resected specimen.

Following surgical consultation, it was concluded that definitive surgical intervention was not immediately justified due to the lack of a clear mass on CT imaging. After discussing the options with the patient, ESD was performed. During the ESD procedure, incision of the mucosa revealed a jelly-like substance (**Figures 3a–c**), raising strong suspicion of a mucinous adenoma or adenocarcinoma. A general surgeon was consulted intraoperatively, and a laparoscopic resection of the ileocecal region and partial ascending colon was subsequently performed (**Figure 3d**).

**Figure 4:**
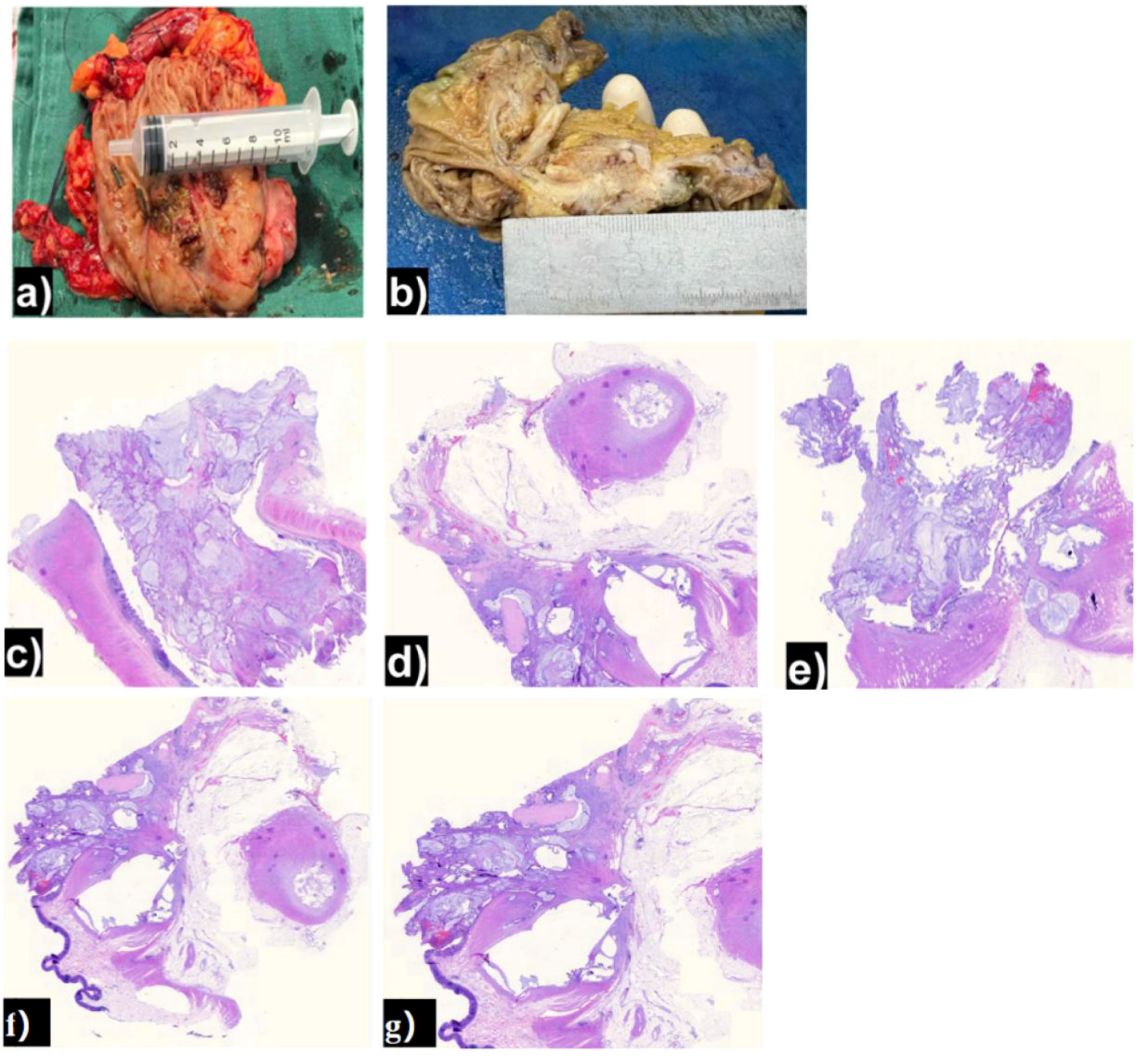
Pathological examination of the resected specimen. **(a, b)** Gross specimen showing the tumor. **(c–e)** Microscopic images (H&E) confirming submucosal mucinous adenocarcinoma with subserosal invasion. **(f, g)** Low-power full-thickness images and high-power images confirm submucosal mucinous adenocarcinoma with subserosal invasion.

**Figure 5:**
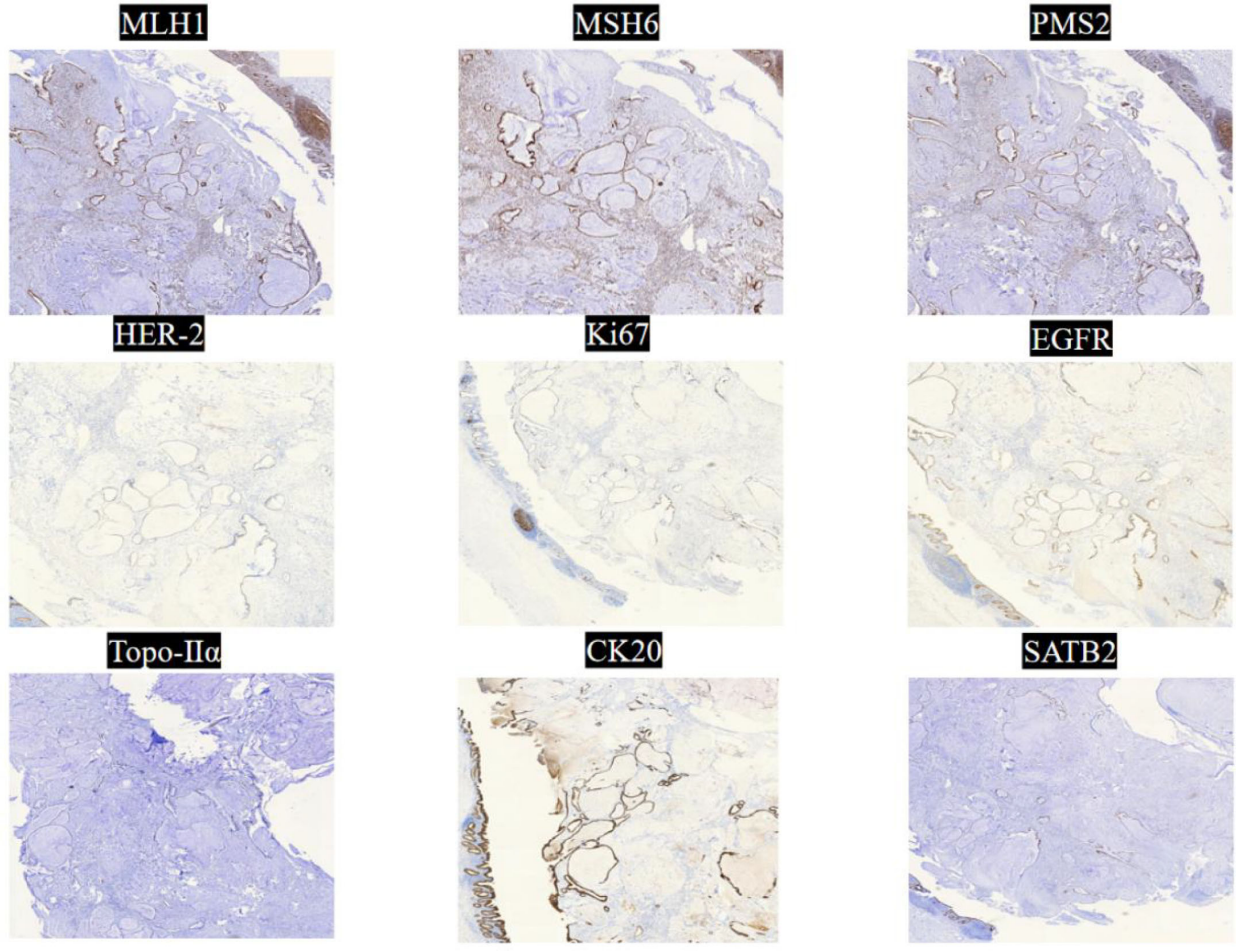
Immunohistochemistry (IHC) results showed: MLH1 (+), MSH6 (+), PMS2 (+), HER-2 (0, with expression), Ki67 (approximately 20% in hotspots), EGFR (focal +), Topo-IIα (+, approximately 20%), CK20 (+), SATB2 (+).

The final pathological examination of the resected specimen (**Figures 4a–g**) was reported as follows: The tumor measured approximately 4.5 × 2.5 × 1.8 cm, infiltrating to the subserosal layer. No evidence of vascular invasion or perineural invasion was identified. The findings were consistent with a mucinous adenocarcinoma originating from the submucosal layer. Immunohistochemistry (IHC) results (**Figure 5**) were as follows: MLH1 (+), MSH6 (+), MSH2 (+), PMS2 (+), HER-2 (Score 0, with expression), Ki-67 (approximately 20% in hotspot regions), EGFR (focal +), Topo-IIα (+, approx. 20%), P53 (moderately positive in approx. 20% of cells), CDX-2 (+), CK20 (+), SATB2 (+), CK7 (-), PAX-8 (-). The subsequent comprehensive pathological and IHC analysis confirmed the diagnosis of a primary colonic adenocarcinoma originating from the submucosa. The patient is currently undergoing adjuvant chemotherapy.

**Figure 6:**
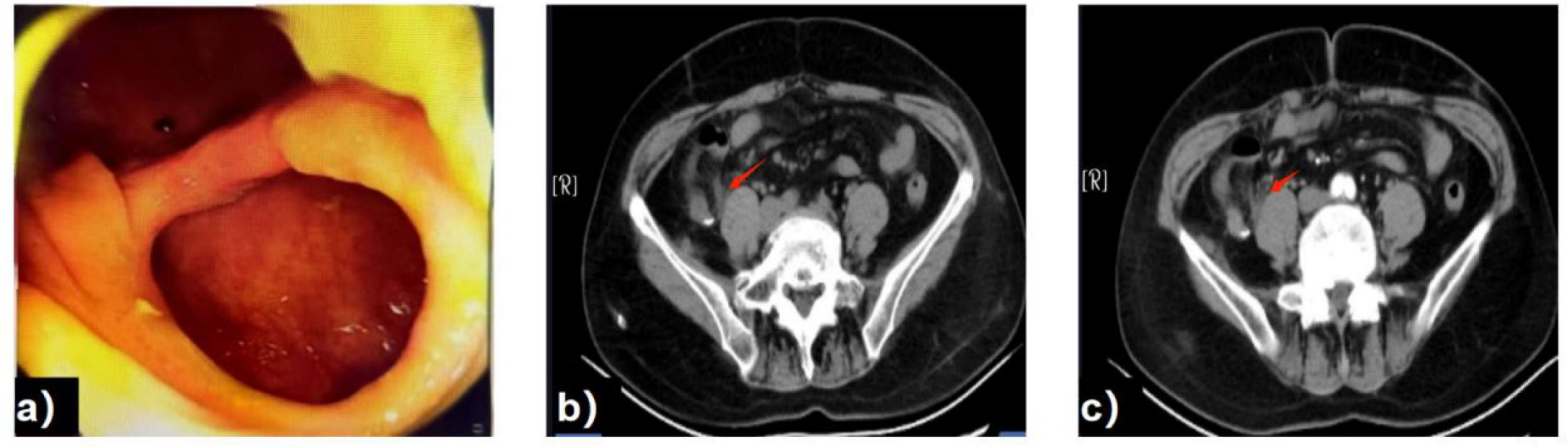
Colonoscopy **(a)** shows Colonic postoperative changes, no abnormalities identified. whole abdominal enhanced CT. **(b, c)** showed no signs of tumor recurrence.

At present, the patient has completed 5 cycles of adjuvant chemotherapy with oxaliplatin combined with capecitabine. After the completion of chemotherapy, re-examination of painless colonoscopy (**Figure 6a**) and whole abdominal enhanced CT (**Figures 6b, c**) showed no signs of tumor recurrence.

## Discussion

This case illustrates a rare presentation of mucinous adenocarcinoma (MAC) of the ascending colon mimicking a submucosal tumor (SMT). Unlike conventional colorectal adenocarcinomas that arise from the mucosal epithelium, MAC is characterized by abundant extracellular mucin occupying more than 50% of the tumor volume, with malignant cells floating within mucin pools (Huang et al., 2021). In some cases, early invasion through the muscularis mucosae into the submucosal layer allows tumor expansion beneath a relatively intact mucosa, thereby creating an SMT-like appearance.

This distinctive growth pattern explains the diagnostic difficulty observed in our patient. Superficial endoscopic biopsies primarily sample the overlying mucosa and may fail to capture submucosal malignant components, particularly when tumor cells are dispersed within mucin lakes. As a result, repeated biopsies in our case revealed only inflammatory changes despite progressive enlargement of the lesion. Such SMT-like colorectal cancers represent an important diagnostic pitfall.

EUS is widely recognized as a valuable modality for evaluating subepithelial gastrointestinal lesions, particularly in determining the layer of origin and differentiating intramural masses from extrinsic compression (Landi and Palazzo, 2009; Kim, 2015). In this case, EUS confirmed that the lesion originated from the submucosal layer. However, although EUS provides high-resolution structural information, it cannot reliably distinguish between benign submucosal tumors and malignant lesions with an SMT-like growth pattern. Therefore, EUS findings should be interpreted cautiously when histology remains inconclusive. Although EUS-guided fine-needle aspiration biopsy has been proposed as an adjunctive diagnostic option in selected SMT-like colorectal lesions, its feasibility in right-sided colonic tumors may be limited. In the present case, ESD provided direct visualization and facilitated timely surgical decision-making.

The pivotal moment in this case occurred during endoscopic submucosal dissection (ESD), when incision of the mucosa revealed abundant gelatinous material. This intraoperative finding strongly suggested a mucin-producing neoplasm and raised immediate concern for malignancy. Given the risk of tumor dissemination and the need for oncologically adequate resection with lymphadenectomy, laparoscopic ileocecal and ascending colon resection was performed during the same anesthetic session. ESD in this context served a diagnostic role by providing direct visualization and facilitating appropriate escalation of treatment, consistent with its established utility in enabling precise histopathological evaluation (Gambitta et al., 2023).

Clinically, this case emphasizes that colorectal lesions presenting with SMT-like morphology, progressive enlargement, and nondiagnostic superficial biopsies should prompt consideration of mucinous adenocarcinoma. While EUS is valuable for anatomical assessment, it does not exclude malignancy. Unexpected intraoperative findings during endoscopic intervention should trigger immediate reassessment to ensure oncologic adequacy.

In conclusion, mucinous adenocarcinoma of the colon may rarely present as an SMT-like lesion and pose substantial diagnostic challenges. An integrated and flexible approach combining EUS evaluation, endoscopic intervention, and timely surgical management can facilitate accurate diagnosis and safe definitive treatment.

## Data Availability

The original contributions presented in the study are included in the article/supplementary material. Further inquiries can be directed to the corresponding author.
